# Four-point vaginal reconstruction versus conventional suturing techniques for the treatment of congenital vaginal atresia: a prospective randomized controlled trial

**DOI:** 10.3389/fped.2025.1600318

**Published:** 2025-10-09

**Authors:** Xianhui Shang, Yuanmei Liu, Zhendong Zhang, Guangxu Zhou, Hongyang Tan, Yingbo Li, Kaiyi Mao, Zhen Luo

**Affiliations:** ^1^ Department of Pediatric Surgery, Affiliated Hospital of Zunyi Medical University, Zunyi, China; ^2^ Department of Pediatric Surgery, Guizhou Children’s Hospital, Zunyi, China; ^3^ Department of Gynaecology, Affiliated Hospital of Zunyi Medical University, Zunyi, China

**Keywords:** congenital vaginal atresia, four-point vaginal reconstruction, conventional suturing, surgical duration, postoperative complications, functional recovery, patient satisfaction

## Abstract

**Background:**

Congenital vaginal atresia is a rare developmental anomaly in which the vagina is partially or completely replaced by fibrous tissue despite normal uterine and ovarian development. Surgical reconstruction is the only effective treatment, and the choice of technique may critically influence outcomes. This study compared the clinical effectiveness of four-point vaginal reconstruction with conventional suturing.

**Methods:**

A prospective, randomized controlled trial was conducted at the Affiliated Hospital of Zunyi Medical University. Eligible patients diagnosed with congenital vaginal atresia were stratified by atresia type (distal, proximal, or complete) and randomly assigned to undergo either four-point vaginal reconstruction or conventional suturing. The predefined primary endpoints were operative duration, postoperative complications, functional recovery, and patient-reported satisfaction.

**Results:**

A total of eighty-three patients were enrolled. Forty-two were assigned to the four-point reconstruction group and forty-one to the conventional suturing group. Operative duration was significantly shorter in the four-point group compared with the conventional group (45.3 ± 12.7 vs. 65.4 ± 15.6 min; *P* < 0.05). No cases of vaginal stenosis were observed in the four-point group, whereas five occurred in the conventional group. Vaginal depth and functional recovery rates were higher in the four-point group, although differences did not reach statistical significance. Patient-reported outcomes, including FSFI domain scores (desire, arousal, orgasm, satisfaction) and SF-36 quality-of-life measures, were consistently higher in the four-point group, with several domains achieving statistical significance (*P* < 0.05).

**Conclusions:**

Four-point vaginal reconstruction may offer clinical advantages over conventional suturing techniques in the surgical management of congenital vaginal atresia. The technique was associated with shorter operative time, fewer complications, and improved functional and patient-reported outcomes. However, as a single-center study with a moderate sample size, these findings warrant further validation in larger, multicenter cohorts.

## Introduction

Vaginal atresia can be classified as congenital or acquired, with congenital cases being more common in clinical practice. It typically manifests as partial or complete vaginal atresia. Vaginal atresia can occur independently or be associated with uterine and urinary system developmental abnormalities (1). The incidence of congenital vaginal atresia is approximately 1 in 4,000 to 1 in 10,000 births (2). Clinically, patients with vaginal atresia are often diagnosed after puberty due to the absence of menstruation or abdominal pain caused by uterine fluid accumulation (3). The condition usually presents as primary amenorrhea accompanied by periodic lower abdominal pain, with symptoms gradually worsening over time. Ultrasound or magnetic resonance imaging (MRI) are effective diagnostic tools for vaginal atresia, providing clear diagnostic information and aiding in surgical planning (4–8).

Surgery is the only effective treatment for congenital vaginal atresia. The timing of the surgery and the choice of surgical techniques significantly impact the treatment outcomes. Although traditional suturing techniques are widely used for treating vaginal atresia, due to the anatomical complexity of the vagina and the limitations of suturing techniques, conventional suturing methods may lead to complications such as vaginal stenosis. This study aims to evaluate the clinical efficacy of four-point vaginal reconstruction vs. conventional suturing techniques in the treatment of congenital vaginal atresia and to explore the advantages of four-point vaginal reconstruction in reducing surgical time, minimizing complications, improving functional recovery, and enhancing patient satisfaction.

This study adopts a prospective randomized controlled trial design to compare clinical outcomes between four-point vaginal reconstruction and conventional suturing techniques in the management of congenital vaginal atresia. The findings suggest that four-point reconstruction may offer advantages in reducing operative time and complication rates, while also potentially enhancing functional recovery and patient satisfaction. These results indicate that the four-point technique could represent a clinically favorable surgical option for selected patients with congenital vaginal atresia, though further validation in larger, multicenter studies is warranted.

## Methods

### Study design and participants

This prospective, randomized controlled trial was conducted between September 2012 and March 2023 to compare the clinical efficacy of four-point vaginal reconstruction with that of conventional suturing techniques in the management of congenital vaginal atresia. Patients with a confirmed diagnosis of congenital vaginal atresia at the Affiliated Hospital of Zunyi Medical University were enrolled. All participants were female adolescents under the age of majority and were observed longitudinally during follow-up. The diagnosis was established on the basis of clinical presentation and confirmed by imaging modalities, including ultrasound, computed tomography, or magnetic resonance imaging. According to the anatomical site of the atresia, patients were classified into three subtypes: distal, proximal, and complete.

Randomization was performed using a computer-generated random number sequence. Allocation concealment was achieved through the use of sequentially numbered, opaque, sealed envelopes (SNOSE), which were prepared and managed by an independent study coordinator.

Due to the nature of the surgical intervention, blinding of the surgeon and patients was not feasible. However, the primary outcome assessors—including those evaluating the FSFI scores and patient satisfaction questionnaires—were blinded to group allocation in order to minimize assessment bias.

Sample size estimation was based on detecting a clinically meaningful difference in the incidence of postoperative vaginal stenosis between the two groups. According to pilot data, the stenosis rate after conventional suturing was estimated at approximately 15%, while the rate following four-point reconstruction was expected to be 0%. Assuming a two-sided α level of 0.05 and a statistical power of 80%, the required sample size was calculated using PASS software.

The study protocol was approved by the Ethics Committee of the Affiliated Hospital of Zunyi Medical University, and written informed consent was obtained from all participants.

Given that all participants were under the age of 18, additional ethical safeguards were implemented. Written informed consent was obtained from the legal guardians of all patients, along with assent from the patients themselves, in accordance with institutional and international ethical guidelines.

For assessments involving sensitive topics such as sexual function (e.g., FSFI questionnaire), participants and their guardians were thoroughly informed of the medical purpose, scientific relevance, and strict confidentiality of the data. The FSFI was administered in a private setting, and participants could opt out at any stage.

To support the psychological well-being of the adolescent patients, certified mental health professionals were available throughout the study period to provide counseling and psychological evaluation as needed, particularly during preoperative counseling and long-term follow-up.

### Inclusion criteria

1. Female patients aged under 18 years;
2. Confirmed diagnosis of congenital vaginal atresia based on clinical and imaging findings (ultrasound, CT, MRI);
3. Initial identification after menarche or due to abdominal pain caused by uterine fluid accumulation;
4. Agreement to participate and provision of informed consent;
5. Minimum follow-up period of 2 years.

### Exclusion criteria

1. Presence of severe comorbidities (e.g., cardiovascular disease, severe liver or kidney dysfunction);
2. Pregnancy or breastfeeding;
3. Malignancy of the vagina, uterus, or adnexa;
4. Withdrawal from the study or incomplete follow-up data;
5. Other chronic conditions severely impacting quality of life.

## Surgical technique

### Preoperative preparation

All surgical procedures were performed by the same senior pediatric surgeon with more than 10 years of experience in pediatric gynecologic surgery. The assisting surgical team remained consistent throughout the study and had undergone standardized training to ensure procedural reliability.

To minimize the potential impact of the learning curve on outcomes, all cases were randomized and evenly distributed between the two study groups in the order of patient enrollment. Prior to trial initiation, the operating team completed standardized training and technical validation of the four-point vaginal reconstruction method. This pre-study preparation ensured procedural uniformity and reduced technical variability across cases.

All patients underwent standardized preoperative preparation. A preoperative metronidazole retention enema was administered to reduce postoperative bacterial contamination, particularly in the perineal region. Prophylactic cephalosporin antibiotics were given intravenously 30 min before surgery to decrease postoperative infection risk. Preoperative imaging assessments, including gynecological ultrasound and magnetic resonance urography (MRU), were performed to determine the location and severity of vaginal atresia.

**Figure 1:**
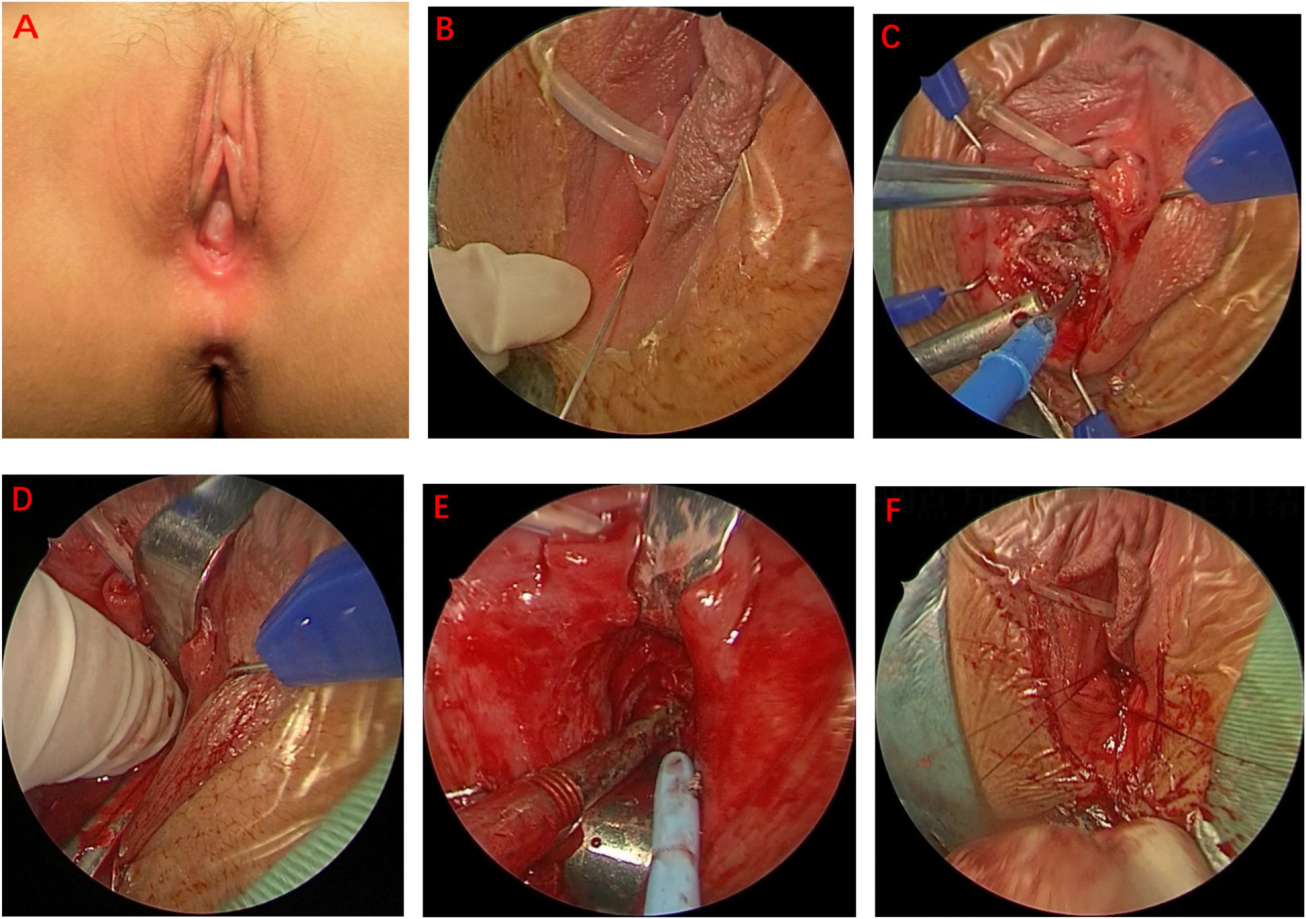
Surgical procedure of four-point vaginal reconstruction for vaginal atresia: **(A)** external appearance of vaginal atresia **(B)** injection of expansion fluid **(C)** cruciate incision of the atretic vaginal orifice **(D)** digital dilation to establish the neovaginal tunnel **(E)** opening the distal segment of the atretic vagina **(F)** vaginal reconstruction by four-point (3, 6, 9, and 12 o'clock) vestibular-to-vaginal suturing.

**Surgical procedure** (Figure 1).

1. **Urinary catheter placement:** A urinary catheter was inserted preoperatively to avoid intraoperative urethral injury.
2. **Injection of expansion fluid:** Expansion fluid was injected to create surgical space and enhance visibility.
3. **Surgical exposure:** The surgical field was exposed using vaginal circumferential retractors. Digital rectal examination was performed to precisely localize the atretic region, followed by changing sterile gloves. An electrosurgical knife was then used to open the atretic vagina, establishing a clear surgical pathway.
4. **Establishing the vaginal tunnel:** A vaginal tunnel was progressively formed using fingers and vaginal dilators to ensure adequate dilation.
5. **Atresia management:** Menstrual blood was aspirated using a syringe. A retained needle was then inserted to precisely identify the atresia site. Saline solution was injected into the uterus through the needle, expanding the uterine cavity and facilitating vaginal mobilization towards the perineum. The atretic area was gradually opened, fluid was aspirated, and the uterine cavity was thoroughly irrigated with saline until clear. A vaginal width of approximately 2.5–3.0 cm, sufficient for two adult fingers, was ensured.
6. **Vaginal reconstruction:** In complete vaginal atresia, a suprapubic incision was performed to access the pelvic cavity, and a neovagina was created using peritoneum. For proximal or distal vaginal atresia, vestibular tissue was utilized to reconstruct the vaginal mucosa. Interrupted absorbable 3–0 sutures were used to suture the vaginal wall and vestibule.
7. **Four-point reconstruction group:** Sutures were precisely placed at 12, 3, 6, and 9 o'clock positions on the vaginal wall to evenly distribute tension. No cases of postoperative vaginal stenosis or severe complications occurred in this group during follow-up.
8. **Conventional suturing group:** Conventional vaginal reconstruction was performed by placing multiple interrupted sutures circumferentially around the vaginal wall. Postoperative management, including the use of drainage and standardized dilation protocols, was identical to that in the four-point reconstruction group.

**Postoperative management:** A 16-Fr drainage tube was placed in the newly reconstructed vagina to facilitate fluid drainage. All patients in both groups received a standardized postoperative vaginal dilation protocol to minimize the risk of vaginal stenosis. An artificial vaginal mold (approximately 2.5–3.0 cm in diameter, constructed from a condom) coated with erythromycin ointment was inserted immediately after surgery and retained *in situ* for 10 days to maintain canal patency. After removal of the mold, patients were instructed to continue dilation training using the same device: once daily for 5 min during the first postoperative month, followed by twice weekly sessions of 5 min each in subsequent months. The dilation protocol, technique, and compliance education were applied uniformly across both surgical groups.

### Assessment of functional recovery

Functional recovery was defined based on a composite of three validated parameters:

(1) Vaginal depth: measured postoperatively using a calibrated vaginal probe by the attending gynecologist.
(2) Vaginal patency: confirmed through physical examination and instrumental assessment, ensuring no evidence of stenosis, adhesions, or obstruction.
(3) Sexual function recovery: evaluated using the internationally validated Female Sexual Function Index (FSFI), a multidimensional self-report instrument developed by Rosen et al., which assesses sexual desire, arousal, lubrication, orgasm, satisfaction, and pain. The FSFI has been translated and validated across various populations and languages (9).

Patient satisfaction was assessed using the Patient Satisfaction Questionnaire (PSQ), a validated tool previously applied in adolescent gynecology studies. The PSQ encompasses domains such as treatment experience, outcome satisfaction, and willingness to recommend (10, 11).

These measures were selected to ensure that both anatomical and functional outcomes were comprehensively evaluated with high reliability and reproducibility.

### Primary and secondary outcomes

Primary outcomes: surgical duration, postoperative complications (wound dehiscence, infection, vaginal stenosis), and functional recovery (vaginal depth and patency).

Secondary outcomes: patient satisfaction and quality of life, assessed by (1) the Patient Satisfaction Questionnaire (PSQ) for overall treatment satisfaction; and (2) the SF-36 Health Survey Questionnaire for general health status and quality of life.

### Follow-up and data collection

Follow-up assessments were conducted at 1, 3, 6, and 12 months postoperatively and after initiation of sexual activity, depending on individual developmental status.

It should be noted that the follow-up time presented in the baseline characteristics represents mean ± SD rather than the exact duration for each patient. The design ensured that all participants reached evaluable developmental stages, while some younger patients underwent longer follow-up to cover outcomes after puberty and sexual maturity. This strategy, while strengthening longitudinal evaluation, may introduce variability across patients and limits external comparability.

Data collected during follow-up included postoperative complications, functional recovery (vaginal depth and patency), patient satisfaction (PSQ), quality of life (SF-36 questionnaire), and sexual function (FSFI).

All assessments were performed using validated instruments administered by trained clinicians or independent evaluators, ensuring consistency and minimizing response bias. In cases where patients were not yet sexually active, FSFI administration was postponed and noted accordingly.

### Statistical analysis

Statistical analyses were performed using IBM SPSS Statistics Professional (version 29.0). Continuous variables were compared using independent *t*-tests or Mann–Whitney *U*-tests, and categorical variables were compared using chi-square tests. A *P*-value < 0.05 was considered statistically significant. Data were summarized using descriptive statistics.

To control for potential confounders, multivariable logistic regression analyses were conducted for primary outcome variables, with adjustments for age, body mass index (BMI), and type of vaginal atresia. Given the variability in patient age, developmental heterogeneity was further examined by stratifying patients into two subgroups based on menarche status. Subgroup comparisons and interaction terms were included in the adjusted models to assess whether surgical outcomes varied with sexual maturity.

For comparisons involving multiple endpoints—such as FSFI subscale scores and SF-36 quality-of-life domains—Bonferroni correction was applied to adjust for multiple testing and reduce the risk of type I error. All statistical tests were two-sided.

## Results

### Clinical characteristics and baseline data

A total of 83 patients with congenital vaginal atresia were enrolled in the study. Patients were categorized into three anatomical subtypes: distal, proximal, and complete atresia. The age at surgery ranged from 9 to 18 years, with a mean body mass index comparable between groups. The median follow-up period was more than seven years across both groups. Among the cohort, 48 patients (57.8%) had experienced menarche at the time of surgery, whereas 35 patients (42.2%) were premenarcheal. Subgroup analysis demonstrated no significant interaction between menarcheal status and surgical type in terms of vaginal patency, functional recovery, or patient satisfaction, suggesting that developmental stage did not substantially alter the primary outcomes. Further analysis showed that differences in anatomical subtypes did not significantly influence the efficacy of four-point reconstruction or conventional suturing. Baseline demographic and clinical features did not differ significantly between the four-point and conventional groups (Table 1).

**Table 1:** Baseline characteristics of study participants.

Characteristics	Four-point group (*n* = 42)	Conventional group (*n* = 41)
Age, mean ± SD (years)	13.2 ± 2.3	13.5 ± 2.1
BMI, mean ± SD (kg/m^2^)	21.3 ± 3.5	22.1 ± 3.2
Follow-up duration (years)	7.5 ± 2.4	7.4 ± 2.6
Atresia type (%)		
Distal vaginal atresia	45%	47%
Proximal vaginal atresia	30%	33%
Complete vaginal atresia	25%	20%

### Surgical outcomes and patient-reported measures

Operative duration was significantly shorter in the four-point group compared with the conventional group (45.3 ± 12.7 vs. 65.4 ± 15.6 min; *P* < 0.05). Postoperative vaginal stenosis occurred in five patients in the conventional group but was absent in the four-point group (0% vs. 6%; *P* < 0.05). Vaginal depth and functional recovery rates were slightly higher in the four-point group (8.3 ± 1.2 cm; 98%) compared with the conventional group (8.1 ± 1.0 cm; 94%), though the differences were not statistically significant (Table 2).

**Table 2:** Comparison of surgical outcomes and patient-reported measures.

Outcomes	Four-point group (*n* = 42)	Conventional group (*n* = 41)	*P*-value
Surgical duration (minutes)	45.3 ± 12.7*	65.4 ± 15.6	<0.05
Postoperative vaginal stenosis (%)	0%*	6%	<0.05
Vaginal depth (cm)	8.3 ± 1.2	8.1 ± 1.0	0.18
Functional recovery rate (%)	98%	94%	0.32
Overall patient satisfaction (%)	95%	92%	0.44
Sexual desire (FSFI)	4.2 ± 0.5*	3.9 ± 0.6	<0.05
Sexual arousal (FSFI)	4.4 ± 0.6*	4.1 ± 0.7	<0.05
Orgasm (FSFI)	4.5 ± 0.5*	4.2 ± 0.6	<0.05
Sexual satisfaction (FSFI)	4.7 ± 0.4*	4.3 ± 0.5	<0.05
SF-36 quality-of-life score	82.5 ± 7.3*	78.2 ± 8.0	<0.05

* *P* < 0.05, representing a statistically significant difference compared with the conventional group.

Patient-reported outcomes further highlighted the benefits of four-point reconstruction. Overall satisfaction was higher, and FSFI scores were significantly superior across multiple domains, including desire (4.2 ± 0.5 vs. 3.9 ± 0.6), arousal (4.4 ± 0.6 vs. 4.1 ± 0.7), orgasm (4.5 ± 0.5 vs. 4.2 ± 0.6), and satisfaction (4.7 ± 0.4 vs. 4.3 ± 0.5; all *P* < 0.05). Quality-of-life assessment by SF-36 also showed a significantly higher total score in the four-point group (82.5 ± 7.3 vs. 78.2 ± 8.0; *P* < 0.05).

### Functional recovery assessment

No cases of vaginal stenosis were observed in the four-point group during follow-up. In contrast, five patients in the conventional group developed postoperative stenosis, all of whom were subsequently managed with dilation until adequate vaginal width was restored. Despite anatomical correction, these patients exhibited slightly lower FSFI scores compared with their counterparts in the four-point group, underscoring the functional advantage of tension-balanced reconstruction.

## Conclusion

This study demonstrates that four-point vaginal reconstruction offers significant advantages over conventional suturing for the treatment of congenital vaginal atresia. The technique shortened surgical duration, reduced postoperative complications, and yielded superior anatomical and functional outcomes, as well as improved patient-reported satisfaction and quality of life. These findings support the four-point technique as a safe, effective, and clinically applicable standard approach.

## Discussion

Congenital vaginal atresia is a rare developmental anomaly in which the vagina is partially or completely replaced by fibrous tissue, despite normal development of the endometrium and ovaries. In certain cases, it may co-occur with cervical dysplasia or anomalies of the urinary and skeletal systems, although such associations are relatively uncommon (12, 13). The embryological basis remains incompletely understood; however, abnormal development of the urogenital sinus is widely considered a central mechanism (14–16).

Clinically, diagnosis is usually made after puberty due to primary amenorrhea or cyclic abdominal pain resulting from hematometra or hematocolpos. Most patients present with progressive pain, normal chromosomal profiles, and preserved gonadal development (17–19). While ultrasonography is commonly used for initial evaluation, it is operator-dependent and susceptible to oversight. Magnetic resonance imaging (MRI) offers superior soft-tissue resolution and has become the preferred modality for preoperative assessment, as it allows accurate identification of the atretic segment, the extent of menstrual blood accumulation, and the degree of outflow obstruction (4–8).

This prospective randomized controlled trial compared the clinical performance of four-point vaginal reconstruction with conventional suturing techniques in patients with congenital vaginal atresia. Our findings indicate that the four-point method was associated with reduced operative duration, fewer complications, improved functional recovery, and higher patient-reported satisfaction. Importantly, subgroup analyses demonstrated that neither menarcheal status nor anatomical subtype (distal, proximal, or complete atresia) significantly modified surgical outcomes, suggesting that the superiority of the four-point approach was consistent across different developmental stages and anatomical variants.

First, four-point vaginal reconstruction significantly reduced operative duration. By anchoring sutures at four standardized sites, tension was evenly distributed along the neovaginal wall, thereby minimizing tissue trauma and simplifying the operative process. In contrast, conventional suturing typically involved more stitches and required intricate adjustment, resulting in longer operative times.

Second, the complication rate in the four-point group was notably lower. No cases of postoperative vaginal stenosis occurred, whereas five instances were reported in the conventional group. Vaginal stenosis is influenced by suture technique, tension distribution, and suture density. By optimizing these parameters through the four-point approach, luminal patency was better preserved. Additionally, the application of a standardized postoperative dilation protocol across all patients likely contributed to the overall low incidence of stenosis and ensured comparability between groups, underscoring the importance of perioperative management in sustaining long-term surgical success.

In terms of functional recovery, patients undergoing four-point reconstruction achieved greater vaginal depth and a higher functional recovery rate. These results underscore the technique's efficacy in restoring neovaginal architecture and function.

Patient-reported outcomes further supported the advantages of the four-point technique. Higher scores were observed across multiple FSFI domains—including sexual desire, arousal, orgasm, and satisfaction—as well as in SF-36 quality-of-life assessments. These improvements suggest that, beyond anatomical reconstruction, the technique may confer significant psychosocial and emotional benefits. The consistency of these outcomes across both anatomical subgroups and developmental stages reinforces the robustness of the findings and strengthens the rationale for broader clinical adoption.

However, several limitations must be acknowledged. First, this was a single-center study with a moderate sample size, which may limit external generalizability. Second, while the follow-up duration was expressed as mean ± standard deviation to reflect overall trends, the actual length of follow-up varied among patients. Younger participants were intentionally monitored for longer periods to ensure that outcomes after entry into puberty and initiation of sexual activity were adequately captured. This design feature enhances the reliability of functional assessments but also introduces heterogeneity that should be considered when interpreting the data. Third, although the follow-up duration was relatively long, extended longitudinal data are still needed to assess sustained outcomes. Third, all patients were adolescents aged 9–18 years at the time of surgery, and none had entered the reproductive stage during the follow-up period. Consequently, vaginal function in relation to fertility and childbirth could not be evaluated. This limitation highlights the need for future studies with longer follow-up into adulthood to assess reproductive outcomes. Finally, while efforts were made to control for confounders such as age and menarcheal status, residual factors—including adherence to dilation training, psychological adaptation, and family support—may have influenced the observed results.

In conclusion, while the four-point vaginal reconstruction technique demonstrated multiple clinical advantages over conventional suturing methods—including reduced operative time, lower complication rates, and superior patient satisfaction—these findings should be interpreted within the context of the study's limitations. The consistent efficacy across different anatomical types and developmental stages suggests broad clinical applicability. Moreover, the deliberate follow-up strategy, which extended into puberty for younger patients but did not yet capture childbirth outcomes, represents both a methodological strength and a critical gap. Standardized postoperative dilation emerges as a critical adjunct in minimizing complications. Future multicenter trials with larger cohorts and longer-term follow-up, extending into patients' reproductive years, are warranted to validate these findings and to clarify the impact of four-point reconstruction on fertility and delivery outcomes.

## Data Availability

The original contributions presented in the study are included in the article/Supplementary Material, further inquiries can be directed to the corresponding author.
